# A growing animal model for neonatal repair of large diaphragmatic defects to evaluate patch function and outcome

**DOI:** 10.1371/journal.pone.0174332

**Published:** 2017-03-30

**Authors:** Mary Patrice Eastwood, Luc Joyeux, Savitree Pranpanus, Johannes Van der Merwe, Eric Verbeken, Stephanie De Vleeschauwer, Ghislaine Gayan-Ramirez, Jan Deprest

**Affiliations:** 1 Department of Development and Regeneration, Katholieke Universiteit Leuven, Leuven, Belgium; 2 Department of Obstetrics and Gynaecology, Prince of Songkla University, Hat Yai, Thailand; 3 Clinical department of Obstetrics and Gynaecology, University Hospitals Leuven, Leuven, Belgium; 4 Department of Pathology, Group Biomedical Sciences, University Hospitals Leuven, Leuven, Belgium; 5 Laboratory Animal Center, Katholieke Universiteit Leuven, Leuven, Belgium; 6 Laboratory of Pneumology, Katholieke Universiteit Leuven, Leuven. Belgium; Faculty of Animal Sciences and Food Engineering, University of São Paulo, BRAZIL

## Abstract

**Objectives:**

We aimed to develop a more representative model for neonatal congenital diaphragmatic hernia repair in a large animal model, by creating a large defect in a fast-growing pup, using functional pulmonary and diaphragmatic read outs.

**Background:**

Grafts are increasingly used to repair congenital diaphragmatic hernia with the risk of local complications. Growing animal models have been used to test novel materials.

**Methods:**

6-week-old rabbits underwent fiberoptic intubation, left subcostal laparotomy and hemi-diaphragmatic excision (either nearly complete (n = 13) or 3*3cm (n = 9)) and primary closure (Gore-Tex patch). Survival was further increased by moving to laryngeal mask airway ventilation (n = 15). Sham operated animals were used as controls (n = 6). Survivors (90 days) underwent chest X-Ray (scoliosis), measurements of maximum transdiaphragmatic pressure and breathing pattern (tidal volume, Pdi). Rates of herniation, lung histology and right hemi-diaphragmatic fiber cross-sectional area was measured.

**Results:**

Rabbits surviving 90 days doubled their weight. Only one (8%) with a complete defect survived to 90 days. In the 3*3cm defect group all survived to 48 hours, however seven (78%) died later (16–49 days) from respiratory failure secondary to tracheal stricture formation. Use of a laryngeal mask airway doubled 90-day survival, one pup displaying herniation (17%). Cobb angel measurements, breathing pattern, and lung histology were comparable to sham. Under exertion, sham animals increased their maximum transdiaphragmatic pressure 134% compared to a 71% increase in patched animals (p<0.05). Patched animals had a compensatory increase in their right hemi-diaphragmatic fiber cross-sectional area (p<0.0001).

**Conclusions:**

A primarily patched 3*3cm defect in growing rabbits, under laryngeal mask airway ventilation, enables adequate survival with normal lung function and reduced maximum transdiaphragmatic pressure compared to controls.

## Introduction

Congenital diaphragmatic hernia (CDH) occurs in 2,6/10,000 live births [1]. Once the initial problems of ventilatory insufficiency and pulmonary hypertension due to pulmonary hypoplasia are managed, the defect requires surgical closure. This can be undertaken primarily for small defects or in the case of larger defects a patch repair may be required. Prenatal lung size predicts for post-natal outcome which is closely linked to defect size[2–4]. As promising *in utero* treatments to accelerate lung growth emerge, a new population of survivors will require a more challenging defect closure[5]. Reported postnatal patch rates in prenatally treated fetuses are around 70%, whereas it is 23% in the population covered by the CDH registry[4,6]. Diaphragmatic patch repairs exhibit significant re-herniation rates [7–9]. With children undergoing patch repair in comparison to those repaired primarily, reporting higher rates of scoliosis (10% vs. 0%), chest wall deformities (14% vs. 6%), small bowel obstruction (12% vs. 6%) and poorer long term pulmonary function testing[10,11]. The contribution of the nature of the patch to these problems is not well understood. None of the available materials mimic the complex muscular-tendinous structure of the diaphragm, which has important roles in both the respiratory system and gastrointestinal tract [12]. To improve long term outcomes, more biocompatible and functional diaphragmatic substitutes are needed, to overcome problems such as recurrence, small bowel obstruction, adhesions and gastro esophageal reflux disease [10]. Most of these problems occur in the first two years of life. This mirrors the time of greatest change in the thorax; by two years the rounded pattern of infancy is superseded by the more ovoid cross sectional shape of adults[13].

Growing animal models including rats, rabbits, lambs, pigs and dogs have been used to mimic the rapidly expanding rib cage in the human[14–18]. Large animal models, particularly the rabbit have the advantage that they are more similar to human in terms of thoracic size, pulmonary function and growth rates[19]. Previously, we tested patch biocompatibility in a growing rabbit model of diaphragmatic hernia[15]. Rabbits are relatively inexpensive, easy to both house and handle and very fast growing, reaching full size by approximatively six months of age [20]. Also, experimental rib fusion at young age induces scoliosis, a condition seen clinically in CDH [21].

As an important respiratory muscle, diaphragmatic replacement can be assessed by its impact on pulmonary function alongside its biomechanical function. In the experimental literature pulmonary outcomes are not frequently reported. As far as we are aware there are limited comprehensive studies analyzing pulmonary function and correlating it to lung histology, hence it remains unclear if pulmonary function is of any value at later time-points [22,23]. Functional evaluation of diaphragmatic replacements includes electromyography (rats) and movement on videoscopic X-ray (dogs, rabbits, pigs) [24–27]. Transdiaphragmatic pressure measurements are used as an index diaphragmatic force and contraction, hence its functional capacity as a respiratory muscle [28]. Transdiaphragmatic pressures have previously been used to assess diaphragmatic re-innervation in rabbits[29].

Herein, we used the young rabbit to develop a more representative animal model for neonatal CDH-repair. Such a model could then later be used for studying novel implants. Firstly, we compared survival rates for different defect size and ventilation modes. Once survival was adequate, we focused on the development of more comprehensive and representative outcome measures, in a controlled study comparing Gore-Tex repaired animals and sham controls.

## Materials and methods

This experiment was approved by the institutional animal ethical committee (KU Leuven P112/2014). To obtain adequate survival there was several phases of this experiment described in detail below: i) tracheal intubation, laparotomy and subtotal left hemi diaphragmatic (HD) excision, ii) tracheal intubation, laparotomy and reduced left hemi diaphragmatic defect size (3*3cm), iii) laryngeal mask airway (LMA) intubation, laparotomy and reduced defect size (3*3cm). Finally, we compared sham operated to Gore-Tex implanted animals.

### Surgery

In this part of the experiment we used in total 30 six-weeks old male rabbits (*Oryctolagus cuniculus*; New Zealand White, Laboratory Animal Center, KU Leuven) to undergo primary repair of a surgically induced left diaphragmatic hernia. They were group housed in a controlled environment. Prior to surgery, animals were weighed, pre-oxygenated, and anesthesia was induced with intra-muscular injection of ketamine 35mg/kg (^®^Ketalar 100mg/ml, Eurovet, Bladel, Netherlands) and xylazine 5mg/kg (^®^XYL-M 2%, VMD, Arendonk, Belgium) into the quadriceps. Simultaneously, they received a subcutaneous injection (SC) (neck) of enrofloxacin 10 mg/kg (^®^Baytril 5%, Bayer, Diegem, Belgium), buprenorphine 0.05mg/kg (^®^Vetergesic 0.3mg/ml, Ecuphar, Oostkamp, Belgium) and meloxicam 0.6mg/kg (^®^Metacam 5mg/ml, Boehringer Ingelheim, Germany). Animals were then placed on a heating pad (Flexiguard 55, Petnap Ltd, Tadley Hants, UK) for the duration of surgery with continuous heart rate and saturation monitoring. A 26 Gauge venflon (^®^BD Neoflon, Erembodegem, Belgium) was inserted into the lateral ear vein and secured (4–0 Vicryl ^®^Ethicon, Johnson and Johnson Medical, USA). Maintenance anesthesia was commenced with an IV bolus of ketamine (1mg/kg) and propofol (2mg/kg) (^®^PropoVet 10mg/ml, Abbott, Maidenhead, UK) followed by a continuous infusion of ketamine (100ug/kg/min) and propofol (0.8mg/kg/min)[30]. Further boluses were given in case of signs of awakening. In the initial animals (n = 27), tracheal intubation was endoscopically guided using a rigid neonatal bronchoscope (10339F) with a 1.3mm fiber endoscope (11540AA; both ^®^Karl Storz, Tuttlingen, Germany). In brief, a semi-rigid guide wire (TSF-35-145, 0.35”, 145cm; ^®^Cook medical) was passed through the vocal cords under endoscopic vision. Over it, a 10Ch (2mm) endotracheal tube was advanced blindly[15]. Due to tracheal complications (as discussed later) subsequent animals (n = 15) were intubated using a laryngeal mask airway (LMA) (^®^V-gel: R2, Millpledge, Wingene, Belgium). The LMA was attached to a mechanical ventilator (Babylog 8000, ^®^Dräger) with standardized ventilator settings: TI 0.4, TE 0.6, Flow 8, Pin 14, PEEP 4–5, 40–50% O2 adjusted according to saturations.

**Fig 1:**
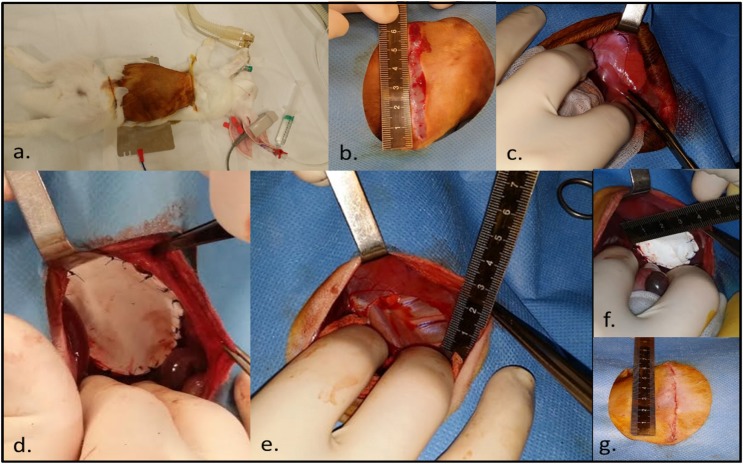
Creation and repair of a left diaphragmatic defect in a 6-week old rabbit. A. Operative position, B. Left subcostal transverse incision, C. Division of the posterior triangular ligament of the liver, D. Subtotal left diaphragmatic excision with Gore-Tex mesh repair, E. 3*3cm diaphragmatic defect, F. Repair with a 3,5*3,5cm Gore-Tex patch G. Sub-cuticular skin closure.

The left upper abdomen and thorax were shaved (^®^Aesculap ISIS, Beringen, Belgium) and the animal secured in the operative position (Fig 1A). Under aseptic conditions local anesthetic was infiltrated (1% lignocaine, AstraZeneca, Brussels, Belgium) at the incision site. Following a left subcostal transverse incision (Fig 1B), sharp and blunt discussion with diathermy (^®^Force2, Valleylab, Dre, Louisville, USA) allowed access to the peritoneal cavity. The liver was gently retracted with a damp gauze permitting division of the left triangular ligament (Fig 1C) with visualization of the left hemi-diaphragm. A diaphragmatic defect was created via excision of the musculo-tendious left hemi-diaphragm. Initially, there was subtotal left hemi-diaphragmatic excision leaving a 1cm antero-postero-lateral and 2cm medial rim (n = 16; Fig 1D). Because of lower than expected survival rates we moved to induction of a smaller yet standardized 3x3cm posterior defect (Fig 1E; n = 11). To repair the defect a dome-shaped 1-mm single layer ^®^Gore-Tex Dual Patch (depending on size: 5cmx3,5 or 3,5x3,5cm) was sutured in place using non-absorbable interrupted suture (Prolene 4–0, Ethicon) (Fig 1F). The laparotomy was closed in two layers (4–0 vicryl) followed by SC skin closure (4–0 Monocryl Ethicon) (Fig 1G). The ketamine/ propofol infusion was slowed and stopped during closure. Removal of the LMA was attempted only when the animal gained consciousness and began to reject the airway. Recovery was in a quiet and warm area with 4L O_2_ via a nose cone. For three days a single daily SC injection of meloxicam and enrofloxacin was administered for pain control. Animals were monitored daily; if they developed respiratory distress O_2_ saturations and clinical examination were undertaken at least twice daily. Low saturations <93% despite oxygen therapy and treatment with antibiotics, or visible discomfort despite adequate analgesia, signified by poor oral intake, reduced movement and weight loss over several days were considered humane endpoints. Subsequently we created 6 “sham” operated controls, who underwent a similar procedure via laparotomy, division of the posterior triangular ligament of the liver and their abdomen packed with damp swabs for 20 mins with no further surgical intervention.

### Outcomes at 90 days

Outcome measures were assessed at 90 days. In case the animal died before 90days a post-mortem examination was undertaken to determine cause, alongside harvesting appropriate histology specimens.

### Chest x-rays prior to harvest

24-48hrs prior to outcome measurements, animals were sedated (IM ketamine: 35mg/kg with xylazine: 5mg/kg as previously) to have posterior-anterior and left lateral chest x-rays (^®^Embrace DM 1000 Mammography System; Agfa-Gevaert, Mortsel, Belgium: collimation size 24x29cm, thickness 280mm, voltage 28 kVp, engine load 65 and 80mAs). Chest x-rays were assessed by an observer blinded to the experimental surgery for re-herniation. To determine the degree of scoliosis the Cobb angle was measured between the 4^th^ and 10^th^ vertebrae in animals with no obvious curvature or at the extremes of the spinal curvature ^(®^RadiAnt DICOM Viewer 3.2.2, Medixant, Poznan, Poland). Measurements were repeated three times and averaged.

### Trans-diaphragmatic pressure measurement and breathing pattern

At 90 days animals were sedated (IM ketamine/ xylazine) and commenced on an IV infusion of ketamine/propofol (as above), with 4L O_2_ given via a nose cone. They were placed supine, neck extended and shaved. A tracheostomy was performed in a stepwise fashion. 1% lignocaine was infiltrated 5mm below the thyroid cartilage on the neck. A 2cm horizontal incision neck incision was made and subplatysmal flaps were elevated with division of strap muscles. The thyroid was gently separated or avoided and pre-tracheal fascia opened. The tracheal cartilage was exposed from the cricoid cartilage to 5^th^ tracheal ring. A small vertical incision between the 2^nd^ to 4^th^ tracheal ring with a 3–0 endotracheal tube (35mm cut short). The tracheal tube was secured by passing two sutures (2–0 Vicryl) around the trachea. The nose cone was moved to cover the tracheal tube.

Measurement of transdiaphragmatic pressure (Pdi) required placement of a pneumotachograph (PTG), intra-thoracic and intra-abdominal pressure catheters. The PTG enabled measurement of tidal volume (Vt) and airflow (ml/s). It was attached to a heater control unit (8411B, Hans Rudolph, Sawnee, KS USA) was attached to the end of the tracheal tube and a pressure transducer (Biopac MP150, Cerom, Paris, France). Two esophageal balloon catheters (5Ch, Cooper surgical, Trumbull, USA) were advanced from the mouth into the stomach (approx. 30cm) and attached to the pressure monitors giving a positive signal. One catheter was retracted until the signal became negative and then assumed to be in the distal esophagus which was synonymous to thoracic pressure. The catheters were labeled esophageal (Peos) or abdominal (Pab) and secured with tape. Once the animal was in an established breathing pattern a run of 5 consecutive breaths was recorded at rest. Then the ET tube (at tracheostomy site) was occluded and 5 attempted breaths recorded. Following these the animal was euthanized with an IV injection of T61 (MSD, Brussels, Belgium).

### Histopathology

Animals were assessed for any signs of incision site breakdown (infection/ dehiscence) before a midline laparotomy was performed. The abdominal cavity was opened and inspected for fluid collections, infection or herniation of the diaphragm. A left thoracic window was also created with removal of the anterior section of the 4^th^ to 6^th^ ribs. The diaphragm was then explanted *en bloc*. Right hemi diaphragmatic specimens were taken from the lateral muscular edge in both sham and Gore-Tex operated animals. The lungs and trachea were removed en bloc and inflated at 20cm H2O with 4% neural buffered formaldehyde (*b*FA), the trachea was tied with a knot and immersed in *b*FA for 24 hours. Random sections were made from the left superior lobe (upper and lower lobe) and from the left inferior lobe (upper and lower). In the right lung a random section was taken from the superior and antero-inferior lobe.

Paraffin blocks were cut into 4μm sections of the lung and right hemi-diaphragmatic specimens and stained with hematoxylin and eosin for airway morphometry and cross sectional area respectively. Microscopic quantification was done by a single observer (MPE) blinded to the experimental group, with a Zeiss light microscope (Axioskop, Carl Zeiss, Oberkochen, Germany) at a magnification of 200x. We assessed the left lower lobe as it is adjacent to the patch border. Each lower lobe was divided into 20 non-overlapping fields with three measurements taken: mean terminal bronchiolar density (MTBD), which is inversely proportional to the number of alveoli supplied by each bronchus, mean linear intercept (Lm) which is related to airspace size, mean wall transection length (Lmw) which is an index of the thickness of alveolar septa[31]. Right-hemi diaphragmatic specimens had the perimeters of at least 100 circular randomly selected fibers delineated and cross-sectional area (CSA) was automatically calculated (Zen 2.3, Carl Zeiss)[32]. This was then grouped by CSA (0–1000μm^2^, 1000μm^2^-2000μm^2^ etc), the percentage of fibers in each group was calculated for total fiber load and a histogram was created for Goretex or sham operated animals.

### Statistics

All procedural information was documented as per the recordkeeping guidelines[33]. Data was analyzed using Prism for Windows version 5.0 (Graphpad software, San Diego, CA, USA). A power calculation based on our previous study (tensiometry results) suggested a repair group size of n = 6 would give 80% power (independent t-test)[15]. Data was checked for normality of distribution using a Kolmogorov-Smirnov test, then presented as a mean with SD or median and IQR. Comparison between groups was done by unpaired students t-test or Mann Whitney test. A p-value <0.05 was considered significant. Survival curves are presented as Kaplan-Meier graphs with group comparsions using a Mantel-Cox test. To compare the CSA of fiber sizes a two-way ANOVA with a Bonferroni multiple comparison test was used.

## Results

### Model creation

#### Complete left hemi-diaphragmatic resection

**Fig 2:**
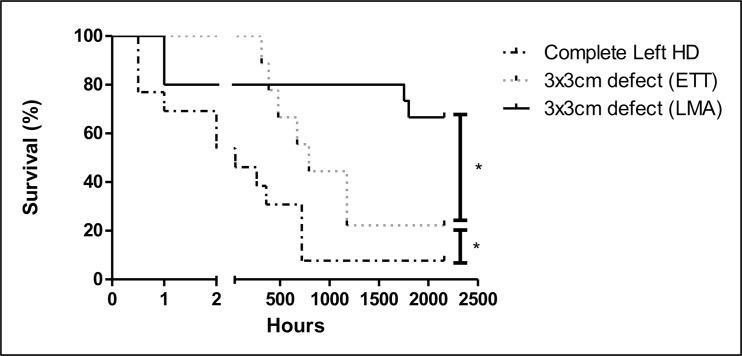
Survival curves. Following a subtotal diaphragmatic excision, a 3*3cm defect with endotracheal intubation and a 3*3cm defect with laryngeal mask airway insertion; all defects were closed with a 3,5*3,5cm Gore-Tex patch* p<0.05.

Initially, 16 rabbits underwent creation of a large defect. Survival at 90 days was 6% (Fig 2). Three (19%) died peri-operatively. One having a gastro-intestinal upset died after induction yet prior to intubation (n = 1). Two others died due to ET tube dislodgement during the operation (n = 2). Therefore, thirteen (81%) survived the operation. In those the resected diaphragm measured either 4,5cm*3cm (10,6cm^3^: 1,3kg (1,13–1,48) n = 4), 5cm*3,5cm (13,7cm^3^: 1,4kg (1,33–1,48) n = 8), 5,5cm*4cm (17,3cm^3^: 1,4kg n = 1). Seven (54%) died <48 hours post-extubation with extreme respiratory distress. Two animals died prior to day 30 (D = 30). Of the six survivors, one died at D11 without obvious cause yet with serous fluid in chest. In a second, pathology confirmed pneumonia (D15). Neither animal had herniation. At D30 (720hrs) three animals were sacrificed due to increasing respiratory distress. One (33%) had a small (∼2cm) re-herniation; lung histology was normal. The second had normal lung pathology, the third diffuse alveolar damage. One animal survived to 90 days without respiratory problems. On post-mortem there was medial herniation of the liver. For this cohort, mean operative time (68±9mins) and weight before surgery (1.40 (1.25–1.45) kg) did not differ between immediate post-operative deaths (<48hours) and those surviving more than 1 week.

#### Reduced defect size with tracheal intubation

**Fig 3:**
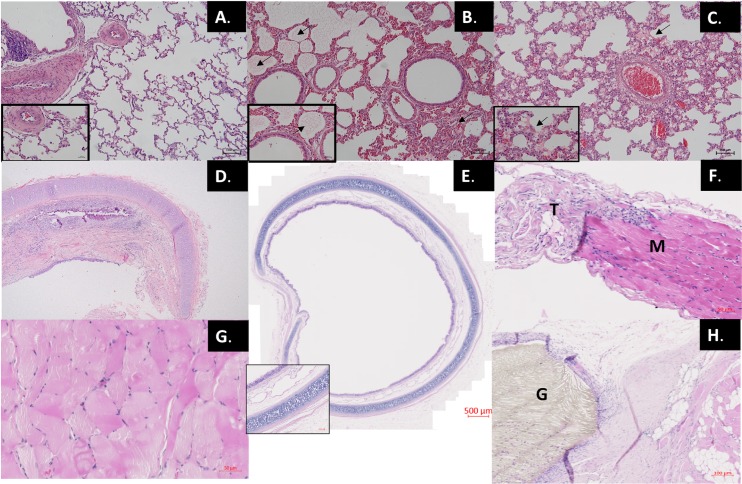
Lung, tracheal and diaphragmatic histology. A. D2: normal lung pathology following a liver haemorrhage (control). B. D16: alveolar and interstitial odema (arrows) following ETT intubation. C. D14: evidence of hyaline membrane formation following ETT intubation. D. D33: Tracheal stricture formation resulting in respiratory distress and death following ETT intubation. E. D90: Intact tracheal muscosa following LMA intubation. F. Untouched 6-week old rabbit diaphragm showing muscle (M) and tendon (T) interface. G. D90: post Gore-Tex implantation right (contra-lateral) diaphragmatic muscle hypertrophy. H. D90: Gore-Tex (G)–tissue interface *D*: *post-operative day*, *ETT*: *endotracheal tube*, *LMA*: *laryngeal mask airway*.

Defect size was then systematically reduced to 3*3cm (7,1cm^3^) to overcome the immediate (<48hours) post-operative mortality (Fig 2). Eleven rabbits were anesthetized, two (18%) died before the operation either during intubation (n = 1) or secondary to iatrogenic pneumothorax (n = 1). No operated animals died within the first 48hrs. Overall, there was an improvement in survival (p <0.05). Of the remaining 9 rabbits, 7 died around D27 (16–49). The majority exhibited significant respiratory distress with significant desaturation (<80%). Post-mortem examination disclosed a small organized hemothorax in one animal. Lung pathology in all but one animal showed edema, respiratory infiltrates and hyaline membrane disease (Fig 3C). This was worse at later time points (Fig 3B and 3C) in keeping with the clinical pattern of respiratory failure. Tracheal pathology revealed strictures with mucosal loss, fibrosis and narrowing of the lumen (Fig 3D) which prompted us to move to working without tracheal intubation (see below). All animals had significant adhesions to the patch. In one animal there was herniation at D = 49. Eventually two out of the remaining nine (22%) survived to 90 days, one with a herniation. Initial weight (1.3±0.5 kg) and operation time (75±11 mins) similar between animals surviving 90 days and those not.

#### Reduced defect size or sham operation with laryngeal mask airway (^®^V-Gel)

To avoid the development of tracheal strictures we moved to securing the airway with an LMA (^®^V-gel). Another nine rabbits underwent standardized induction of a reduced size defect (3*3cm) and primary repair with Gore-Tex to complete the envisaged number of survivors in that group. We then also added six sham operated animals. With this ventilation strategy overall survival was improved to 67% (Fig 2, p <0.05), no sham operated animals died. Notably, after 48 hours post-operatively there were no further episodes of respiratory distress suggestive of tracheal injury. There was normal tracheal architecture at D90 in these animals (Fig 3E). Four (44%) Gore-Tex rabbits survived to 90 days with three early post-operative deaths, all in the Gore-Tex group. Two had confirmed aspirations on post-mortem and no cause of death was found in the other. There were two late deaths at D73 (diarrhea) and D75 (pneumonia). No Gore-Tex repairs had recurrence.

### Gore-Tex implanted compared to sham operated

**Fig 4:**
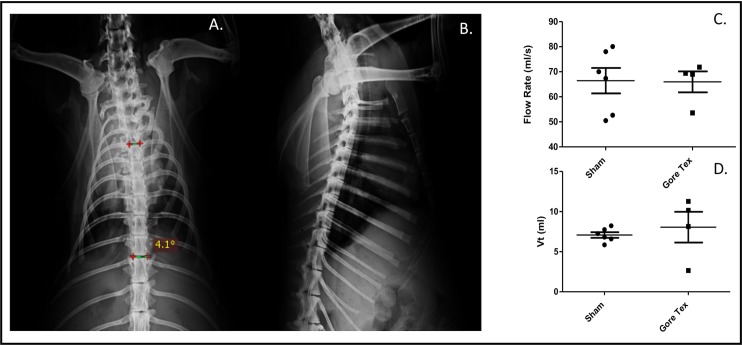
Pulmonary Outcomes. Chest X-Ray (CXR) showing A. Cobb angle measurement (PA film) B. lateral view in sham operated animals and pulmonary function testing showing C. Flow rate, d. Tidal volume.

We compared sham (n = 6) and Gore-Tex (n = 6) implanted animals at 90days. During the experiments, animals doubled in size (weight increase in Gore-Tex animals (220±54%) vs. sham (140±73%; p = 0.0571). In the Gore-Tex group, one had a re-herniation (17%) and one had bilateral pleural effusions, yet without wound infection, dehiscence and unremarkable lung pathology. Chest X-rays at 90 days revealed comparable Cobb angles (sham 3.8°±0.5 vs. Gore-Tex 3.2°±0.8; p = 1.0) (Fig 4A and 4B).

### Trans-diaphragmatic pressure and breathing pattern measurements

**Fig 5:**
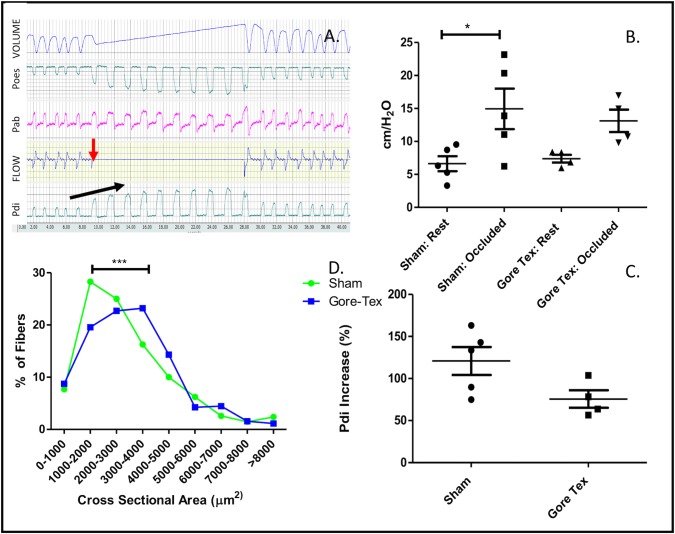
Diaphragmatic Outcomes. A. Transdiaphragmatic pressure measurement: airflow recording showing tracheostomy occlusion with halt of airflow (red arrow), increase in Pdi (black arrow). B. Pdi increases in Sham and Gore-Tex implanted animals following tracheostomy occlusion, C. Increase in Pdi as a % change at rest and following occlusion, D. Right hemi diaphragmatic muscle fiber cross sectional (μm^2^) area is increased in Gore-Tex implanted animals. *Poes*: *oesophageal pressure*, *Pab*: *abdominal pressure*, *Pdi*: *transdiaphragmatic pressure*.

Sham and Gore-Tex rabbits had similar Vt (7.1ml ± 0.8) vs. 8.1ml ± 3.8) and flow rates (66.4*ml/s* ±12.5 vs. 66.*0ml/s* ±8.4) (Fig 4C and 4D). Transdiaphragmatic pressure measurement were feasible in nearly all (Fig 5A and 5B). Tracheostomy occlusion resulted in an increase in Pdi (Fig 5C). At rest (i.e unoccluded), Pdi was similar in sham and Gore-Tex animals (6.6±2.5 mmHg vs. 7.4±1.2 mmHg; p = 0.9048). There was no correlation between tidal volume and Pdi (data not shown). On occlusion of the tracheostomy tube transdiaphragmatic pressure increased in sham operated animals, whereas it did not in Gore-Tex implanted animals (p<0.05, Fig 5B). Overall, following occlusion sham animals increased their transdiaphragmatic pressure 134% as compared to a 71% increase in Gore-Tex operated animals (Fig 5C).

### Histopathology

Lung histology was not different between groups, not for the Lmw (16.9±2.9 vs. 17.0±0.9), neither for the Lm (58.0±4.7 vs, 57.6±5.1) or MTBD (0.80±0.1 vs. 1.0±0.1) (sham vs. Gore-Tex). Pleural thickening was noted in Gore-Tex implanted animals, likely secondary to friction. Contra-lateral diaphragmatic muscular fiber size was increased in Gore-Tex implanted animals (Fig 5D; p<0.0001) (Fig 3G). Representative images include an untouched 6-week old rabbit diaphragm (muscle-tendon interface) and the Gore-Tex diaphragmatic interface (Fig 3F and 3H).

## Discussion

Herein, we improved a large diaphragmatic hernia repair animal model by increasing diaphragmatic defect size to one that could not be directly closed with permissible survival, and by changing ventilation technique. We implanted the most commonly used patch for defect repair; Gore-Tex. This did not induce scoliosis although there was a 17% herniation rate with changes in transdiaphragmatic pressures and compensatory contralateral diaphragmatic hypertrophy.

To increase diaphragmatic defect size to the extent where primary closure was impossible required several modifications to our existing anesthetic protocol[15]. Initially, a large defect despite mechanical ventilation resulted in the majority of animals dying in the immediate post-operative period. As rabbits are diaphragmatic breathers, we speculated that they were unable to compensate for the near complete left hemi diaphragmatic replacement with a patch[34]. A reduction in defect size to around what is categorized as a type B defect (CDH study group staging) improved our immediate post-operative losses (<2hrs)[35]. However, we then ran into the problem of later losses due to tracheal stricture formation. Several models have been used to produce tracheal strictures in rabbits; one implanting an endotracheal tube wrapped in ^®^Surgicel into the tracheal for one week[36]. Another study related tracheal stricture formation to the duration of intubation; with intermittent tracheal trauma throughout the intubation. However, it was only following six hours of trauma that they could routinely produce respiratory distress and stricture formation[37]. Our operative time of around 75mins produced significant and reproducible damage within 2–3 weeks. Despite intubating under direct vision using a guide wire to minimize trauma there are several possible contributors to the damage. In our previous study, with an identical intubation technique we had 19% mortality within two weeks of surgery, the cause of which was unclear. In this study we lost 54% at an intermediate time-point exhibiting significant respiratory distress secondary to tracheal stricture formation. Although we often re-used tracheal tubes rendering them stiffer and more likely to induce damage, these were uncuffed. This combined with the vulnerable immature rabbit airway may explain some of the lost in both of these experiments. The increased mortality may be due to the additional use of mechanical ventilation with positive pressure possibly further exacerbating the injury through mechanical injury. Furthermore, our larger defects may have meant that this tracheal insult was less well tolerated than in the previous experiment. Regardless, these problems were circumvented by the use of a LMA.

We also measured the transdiaphragmatic pressure to evaluate patch repair in the setting of CDH, which is to our knowledge has not been previously reported. Clinically, children with CDH have poorer exercise performance and respiratory function tests, demonstrating obstructive airways [38][39]. The most significant contributor must be the degree of pulmonary hypoplasia but the contribution of the diaphragmatic repair has not been so well considered. We demonstrated a change in diaphragmatic force with a Gore-Tex patch implant with no accompanying change in lung histology. Indeed, patch closure is a predictor for a worse outcome in pulmonary function testing[11,40]. Gore-Tex does not permit significant tissue remodeling whereas a structure which actively encourages tissues ingrowth and re-innervation of the bridging tissue may in time lead to a better functional result[15]. Regardless, in our model the de-innervated area of diaphragm replaced by the Gore-Tex patch will never function as well as native tissue. The phrenic nerve in children with a repaired diaphragmatic defect has been shown to have a prolonged latency and twitch diaphragmatic pressure on the affected side representing a further challenge to the ideal repair[41,42].

This model does not reproduce the primary pulmonary deficit present in CDH survivors. Of note is that rabbits alveolarize in the first few weeks after birth and by the time of patch implant lung development is already complete[43]. Any pulmonary dysfunction would therefore be considered secondary to the surgery or its complications. We found that measurements of breathing patterns confirmed by airway morphometry was relatively unaffected, both in treated and control animals. Although, herniation may lead to atelectasis and lung compression it is unlikely to modify alveolar structure. Previously, in adult rats direct closure of a muscular diaphragmatic defect reduced forced vital capacity (FVC), forced expiratory volume (FEV1) and elastic properties of the lungs significantly within 90 minutes compared to Gore-Tex closure[22]. It is to be expected that a tight primary repair leads to early ventilatory changes however these are likely compensated for in time. Regardless, we confirm here that the patch in itself does not impact respiratory function.

Re-herniation rates in humans vary between centers and it has been reported in the most expert hands they are actually relatively low[8,10]. We report a (re-)herniation rate of 20%, essentially only in one animal, this is likely reflective of the human situation. Although we did not identify any scoliosis, in previous animal models high rates of scoliosis are reported when the patch is secured directly to the ribs, which was not the case in this experiment [14]. It is likely that the aetiology of scoliosis is more complex than we can reproduce in this model. A reduced ipsilateral thoracic size resulting from the restrictive/ obstructive ventilatory defect, excessive tension from the diaphragmatic repair or a smaller lung volume necessitating a smaller thoracic size due to changes in the recoil pressure of the lung all may contribute[44,45].

Finally, we investigated whether there was a compensatory change in the right hemi-diaphragm in response to the left sided intervention. We speculated that the right hemi-diaphragm may hypertrophy in response to increased effort due to the lack of movement in the left and this was confirmed by measuring muscle fiber CSA. Rabbits are known to have around 20% type 1 fibers in their diaphragm and this differs according to region[46]. Under increased strain there is a documented muscle fiber switch from type 2b to type 2a and this could act as another compensatory mechanism[47]. An investigation of muscle fiber type could provide more insight into this disease model, yet we did not pursue this.

To further this study, one could also consider more extensive and non-invasive pulmonary function testing using whole body plethysomography[48]. Furthermore, fluoroscopic x-ray could provide interesting information on ipsilateral and contra-lateral diaphragmatic movement following diaphragmatic patch repair[26]. We also acknowledge that we have compared animals with different ventilation strategies and failed to report the passive biomechanical properties of our diaphragmatic repair. Finally, it would be interesting to explore the host cellular response to implants. This would be most valuable with additional examination of implants at earlier time-points.

Regardless, we present a more comprehensive model for neonatal diaphragmatic hernia repair with novel readouts for testing patch repairs. Future studies in this model could now explore novel solutions for diaphragmatic repair.

## Supporting information

S1 Minimal Dataset Excel file containing all raw data included in Fig 2, Fig 4C and 4D and Fig 5B, 5C and 5D. (XLSX)
